# Strategies to Improve Recruitment to a De-escalation Trial: A Mixed-Methods Study of the OPTIMA Prelim Trial in Early Breast Cancer

**DOI:** 10.1016/j.clon.2020.01.029

**Published:** 2020-06

**Authors:** C. Conefrey, J.L. Donovan, R.C. Stein, S. Paramasivan, A. Marshall, J. Bartlett, D. Cameron, A. Campbell, J. Dunn, H. Earl, P. Hall, V. Harmer, L. Hughes-Davies, I. Macpherson, A. Makris, A. Morgan, S. Pinder, C. Poole, D. Rea, L. Rooshenas, the OPTIMA Prelim Study Group

**Affiliations:** ∗ Population Health Sciences, University of Bristol, Bristol, UK; † National Institute for Health Research, University College London Hospitals Biomedical Research Centre, London, UK; ‡ Warwick Medical School, University of Warwick, Coventry, UK; § Ontario Institute for Cancer Research, Toronto, Ontario, Canada; ‖ The University of Edinburgh, Cancer Research UK Edinburgh Centre, Western General Hospital, EH4 University Cancer Centre, University of Edinburgh, Edinburgh, UK; ¶ Oncology Centre, Addenbrooke's Hospital, Cambridge, UK; ∗∗ Imperial College Healthcare NHS Trust, Charing Cross Hospital, London, UK; †† Beatson West of Scotland Cancer Centre, Glasgow, UK; ‡‡ Mount Vernon Cancer Centre, Mount Vernon Hospital, Northwood, UK; §§ Independent Cancer Patients' Voice, London, UK; ‖‖ King's College London, Comprehensive Cancer Centre at Guy's Hospital, London, UK; ¶¶ Arden Cancer Centre, University Hospitals Coventry and Warwickshire, Coventry, UK; ∗∗∗ School of Cancer Sciences, University of Birmingham, Birmingham, UK

**Keywords:** Breast cancer, de-escalation trial, equipoise, qualitative research, randomised controlled trials, recruitment

## Abstract

**Aims:**

De-escalation trials are challenging and sometimes may fail due to poor recruitment. The OPTIMA Prelim randomised controlled trial (ISRCTN42400492) randomised patients with early stage breast cancer to chemotherapy versus ‘test-directed’ chemotherapy, with a possible outcome of no chemotherapy, which could confer less treatment relative to routine practice. Despite encountering challenges, OPTIMA Prelim reached its recruitment target ahead of schedule. This study reports the root causes of recruitment challenges and the strategies used to successfully overcome them.

**Materials and methods:**

A mixed-methods recruitment intervention (QuinteT Recruitment Intervention) was used to investigate the recruitment difficulties and feedback findings to inform interventions and optimise ongoing recruitment. Quantitative site-level recruitment data, audio-recorded recruitment appointments (*n* = 46), qualitative interviews (*n* = 22) with trialists/recruiting staff (oncologists/nurses) and patient-facing documentation were analysed using descriptive, thematic and conversation analyses. Findings were triangulated to inform a ‘plan of action’ to optimise recruitment.

**Results:**

Despite best intentions, oncologists' routine practices complicated recruitment. Discomfort about deviating from the usual practice of recommending chemotherapy according to tumour clinicopathological features meant that not all eligible patients were approached. Audio-recorded recruitment appointments revealed how routine practices undermined recruitment. A tendency to justify chemotherapy provision before presenting the randomised controlled trial and subtly indicating that chemotherapy would be more/less beneficial undermined equipoise and made it difficult for patients to engage with OPTIMA Prelim. To tackle these challenges, individual and group recruiter feedback focussed on communication issues and vignettes of eligible patients were discussed to address discomforts around approaching patients. ‘Tips’ documents concerning structuring discussions and conveying equipoise were disseminated across sites, together with revisions to the Patient Information Sheet.

**Conclusions:**

This is the first study illuminating the tension between oncologists' routine practices and recruitment to de-escalation trials. Although time and resources are required, these challenges can be addressed through specific feedback and training as the trial is underway.

## Highlights

• Oncologists' routine practice complicated recruitment to a breast cancer trial.
• Oncologists selectively approached eligible patients, informed by routine practices.
• Use of routine language undermined equipoise in discussions with eligible patients.
• Negotiating clinical and research roles can be challenging in de-escalation studies.
• Recruitment challenges were overcome through ‘real-time’ investigation and feedback.

## Introduction

Multiparameter tumour gene expression assays have driven new approaches to adjuvant chemotherapy decision-making for breast cancer [[1], [2], [3]]. The UK's National Institute for Health and Care Excellence has approved the use of several multi-parameter assays (MPAs) to guide adjuvant chemotherapy decisions in patients with oestrogen receptor-positive, human epidermal growth factor receptor 2-negative lymph node-negative breast cancer. Although there is potential to extend this recommendation to node-positive patients, the National Institute for Health and Care Excellence has called for robust randomised controlled trial (RCT) evidence to inform future guidance [4]. For more than 50 years, RCTs have been driving important developments in the treatment of breast cancer [[5], [6], [7]], but recruitment issues have threatened the timely and successful completion of trials [8,9]. This is particularly true of de-escalation of treatment studies, which have come to the fore in early breast cancer research in recent years [10]. Reported obstacles have included logistical issues, lack of eligible patients, limited research resources and patient preferences for/against treatments [11]. Research has revealed a number of challenges for recruiters, including the emotional burden of recruitment, lack of equipoise and reconciling the roles of clinician and researcher [12,13].

The Optimal Personalised Treatment of early breast cancer using Multi-parameter Analysis (OPTIMA) trial [14,15] is a UK-based multicentre clinical trial that randomises patients to adjuvant chemotherapy (current care) versus ‘test-directed’ adjuvant chemotherapy or ‘no chemotherapy’. OPTIMA is designed to show the non-inferiority of this approach to patient outcome, particularly for those with node-positive disease. OPTIMA Prelim was the feasibility phase of the study and was designed to show the acceptability of a large-scale trial of MPA-directed treatment to patients and clinicians. An overview of OPTIMA Prelim is reported in full elsewhere [15,16] and included in the Supplementary Material. The OPTIMA Prelim trial management group (TMG) anticipated that recruitment would be difficult, as many patients who would be offered chemotherapy in current UK practice, would be allocated to ‘no chemotherapy’ within the trial. The TMG therefore integrated the ‘QuinteT Recruitment Intervention’ (QRI) [17] into the RCT protocol. This is a complex intervention that aims to identify and understand sources of recruitment difficulty rapidly, and then develop strategies to address these throughout the remainder of the trial. An evaluation of the effectiveness of the QRI approach using before and after data for several trials, including OPTIMA Prelim, has been published elsewhere [18].

This paper reports on the sources of recruitment difficulty in this challenging trial and the specific strategies implemented to try to overcome these.

## Materials and methods

### Design

This paper reports a mixed-methods study using the QRI [17,19] to understand and address recruitment issues. The QRI consisted of two iterative phases. Phase 1 aimed to understand recruitment obstacles rapidly, using mixed (predominantly qualitative) methods. Phase 2 involved working collaboratively with the chief investigator and TMG to devise and deliver tailored strategies to address recruitment difficulties, based on evidence from phase 1.

The QRI was integrated within the study protocol; ethical approval was granted by the South East Coast–Surrey Research Ethics Committee (12/LO/0515, 22 June 2012).

### QuinteT Recruitment Intervention phase 1: understanding recruitment obstacles

Phase 1 began when recruitment had started. All sites open at the time (*n* = 25) were invited to take part; nine agreed to participate in one or more aspects of the data collection discussed below.

Key informants, including oncologists, research nurses and TMG members, were purposefully sampled for interview based on their role in overseeing or conducting recruitment. In total, 32 staff were invited to participate in in-depth semi-structured interviews about their perspectives on recruitment. Interviews were conducted face-to-face or via telephone (by LR and JLD) and lasted between 40 and 90 min. Research nurses were also approached to take part in shorter, structured interviews regarding the operation of recruitment processes at their sites. Sampling for interviews concluded when data saturation was reached (i.e. when no new issues arose from two consecutive interviews).

Recruiting staff (oncologists and research nurses) were asked to routinely audio-record discussions concerning OPTIMA Prelim with eligible patients (‘recruitment appointments’), including when they accepted or declined trial participation. Initial verbal and subsequent written patient consent was obtained for all audio-recordings.

All interviews and audio-recorded appointments were analysed thematically using the constant comparative method derived from grounded theory methodology [20]. In addition, techniques adapted from conversation analysis [21] were used to examine the structure and sequencing of interactions. All transcripts of interviews/appointments were analysed by one researcher using the above techniques (LR). To enhance reliability, another researcher (SP) analysed 10% of interviews and appointments in the early stages of data collection, with semantic differences in coding discussed and resolved. Going forwards, key findings and interpretations from the analysis were interrogated by the study team (LR, SP, JD) through testing claims with raw data and discussing in regular meetings. Finally, a new researcher (CC) joined the team once data collection was complete and listened to all audio-recorded consultations and reviewed interview transcripts/coding, with a view to agreeing the core messages pertaining to this paper with LR and JD.

The data collection techniques were supplemented with content analysis of patient-facing documentation and descriptive analyses of recruitment screening logs. Screening logs were regularly assessed to summarise the numbers of patients recorded as eligible and the proportion of those who accepted/declined an invitation to participate. Figures were compared over time and across sites.

### QuinteT Recruitment Intervention phase 2: developing and delivering a ‘Plan of Action’ to optimise recruitment

Data and emerging findings from the analyses were considered in tandem to triangulate data and crystallise the key issues that seemed to be undermining recruitment. These issues were reported to the chief investigator/TMG and informed a ‘plan of action’ to optimise recruitment. A statistical evaluation of the impact of these strategies on recruitment has been reported elsewhere [18].

## Results

### Phase 1: understanding recruitment obstacles

Early in the recruitment phase, 14 semi-structured interviews were conducted with TMG members and oncologists from six sites and eight research nurses took part in structured interviews. The interviews were conducted with staff from sites considered ‘mid-range’ in terms of recruitment rates (recruiting 0.32–1.85 patients per month, compared with a range of 0–2.20 in the overall trial). Between November 2012 and January 2014, 46 appointments were audio-recorded, involving 11 oncologists and 29 patients (Table 1). The audio-recordings and interviews were obtained from recruiting sites from Scotland and the South West, South East and Midlands regions of England.

**Table 1:** QuinteT Recruitment Intervention sample and data set

OPTIMA Prelim site number	Semi-structured interviews with health care professionals	Audio-recordings of recruitment discussions between oncologists and patients		
		Number of audio-recordings	Number of patients	Number of oncologists
Site 1	1 oncologist∗ 1 surgeon 1 research nurse	4	2	1 oncologist
Site 2	1 oncologist∗	23	11	4 oncologists
Site 3	4 oncologists 1 research nurse	7	5	2 oncologists
Site 4	0	6	5	1 oncologist
Site 5	0	4	4	2 oncologist
Site 6	0	2	2	1 oncologist
3 non-recording sites	2 oncologists∗ 2 research nurses, 1 trial manager	NA	NA	NA
Total	14	46	29	11

∗ Member of the Trial Management Group.

#### Challenges to recruitment

Analysis of interviews highlighted that oncologists were generally very committed to OPTIMA's endeavour of better targeting which patients benefited from chemotherapy provision and often emphasised the need for evidence-based approaches to reduce the burden of over-treatment. Despite this enthusiasm, difficulties arose at two key points of the recruitment process: approaching eligible patients and discussing OPTIMA Prelim in recruitment appointments.

##### Approaching eligible patients

Discomfort with aspects of the eligibility criteria, coupled with some hesitancy about the accuracy of multiparameter testing resulted in some eligible patients not being approached about trial participation. Oncologists and other members of the multidisciplinary team (MDT) had variable thresholds of comfort regarding different aspects of the eligibility criteria associated with risk of recurrence. This was particularly evident in relation to patients' nodal status. The eligibility criteria allowed for patients with up to nine involved lymph nodes to be included in the study, but oncologists' individual thresholds for permitted nodal involvement varied.

*The only thing that surprised me with the criteria is the lymph node status; one, because it's almost ingrained in our practice and certainly as oncology trainees as we go through doing the exams and so on, that lymph node positivity is a risk factor for a recurrence, and that those patients who are highest risk, benefit from chemotherapy most. (Oncologist Site 4)*

Some oncologists took into account tumour characteristics and criteria not specified in the trial protocol in their judgements of who to approach for the trial. These were criteria that they would usually consider in treatment decisions in routine practice where MPA testing was not normally available. For example, some oncologists were reluctant to approach patients with grade 3 tumours, on the basis that they felt that these individuals would require chemotherapy.

*I think people feel very uncomfortable about letting heavily node positive grade 3 disease [into the trial] … there's something about - they strongly feel those patients need chemo, and the idea that they might go into a trial where they're not being given chemo makes them feel very uncomfortable. (Oncologist Site 2)*

Assessments about patients' emotional state also factored into decisions about approaching eligible patients. Some oncologists and research nurses described instances where patients had been deemed eligible by the MDT but were not approached because of how they presented emotionally at their appointment, and other instances where patients were approached with hesitancy, based on judgements about their ability to cope with trial participation decisions.

*So we might, if we thought they weren't really suitable, we might say “Well, we do have a trial that would help us to choose whether to have chemotherapy or not and would you like us to explain that to you?” in a kind of very general way, and kind of probably hoping that the patient will say “No, I don't want anything like …. oh no I don't want that.” (Research Nurse Site 2)*

##### Discussing OPTIMA Prelim in recruitment appointments

The audio-recorded recruitment discussions revealed how oncologists' communication practices could undermine trial recruitment, despite their best intentions to recruit. Applying routine approaches to discussing adjuvant chemotherapy did not align with the trial's premise, and created problems in some cases, as outlined below.

#### Structure of appointments and conveying chemotherapy uncertainty

All recruiters successfully mentioned uncertainty around the benefits of chemotherapy as part of the RCT rationale. Oncologists generally used one of two approaches to structuring the discussion: (i) introducing chemotherapy as a beneficial treatment (as per routine practice), and then explaining the trial rationale; or (2) introducing the uncertainties around chemotherapy from the outset of the consultation, followed by an explanation of the trial. The first approach sometimes coincided with patients settling on the idea of needing chemotherapy; to then introduce the idea that they may not benefit from chemotherapy and could potentially forego this in OPTIMA Prelim, was therefore somewhat confusing.

Oncologist: *Back to drugs. The other thing that we have to consider, and it's I would say a case of icing on the cake, but nevertheless, we should talk about it, is the benefits of a course of chemotherapy.*

Patient: *Would you recommend it then?*

Oncologist: *I think with what you have, there is probably enough benefit for it to be worthwhile. It should be considered quite seriously.*

[Later]

*Patient: So without chemotherapy?*

Oncologist: *There is a bigger risk that this comes back in the future. It's not a huge risk, but it's enough to make the benefits of chemotherapy worthwhile.*

Patient*: Okay. I've got to do it then, haven't I?*

Oncologist: *There is a possible way out of this.* [Introduces OPTIMA Prelim and the Oncotype DX test].

Patient: *You've completely confused me now. OK, so I accept that I've got to have the chemotherapy…*

By contrast, opening the discussion with the uncertainties of chemotherapy benefit provided a smoother route to explaining the trial rationale.

Oncologist: *The thing that is a little harder to decide is whether you need chemotherapy or not.*

Patient: *It's the big one yeah.*

Oncologist: *It's a harder decision for two reasons. One is, that it’s, lots of patients like yourself would be fine even if they didn't have it, and also it has obviously more side effects [*explains probability of benefit*]. That means though that 90 of them or more who have had chemotherapy have had no benefit.*

Patient: *It's just whether I'm one of those ones.*

#### Conveying equipoise

Participation in OPTIMA Prelim required patients to be happy (in principle) with the idea of being allocated ‘current care’ (chemotherapy) or test-directed treatment, which carried the possibility that they would not receive chemotherapy. As recruiters, oncologists and research nurses needed to convey equipoise by finely balancing the advantages and disadvantages of each of the trial arms. One aspect that proved difficult was addressing uncertainty about the Oncotype DX test's effectiveness, which underpinned the need for the trial. Oncologists and research nurses discussed experiences of some patients opting to access the test outside the National Health Service and other patients not being comfortable with the uncertainty around the test. Conveying the potential benefits and disadvantages of the test proved a delicate balancing act.

Oncologists' language during recruitment appointments had the potential to tip the balance towards or against test-directed treatment. Some recruiters reverted to terms and phrases that would usually be helpful for explaining the rationale for chemotherapy (in routine practice), but this undermined the need to convey equipoise in the RCT context. For example, referring to chemotherapy as ‘icing on the cake’ had the potential to influence a patient's perception of chemotherapy as a worthwhile treatment.

Some oncologists continued their routine practice of referring to online predictor tools (e.g. PREDICT^1^ and Adjuvant! Online^2^) to show likely chemotherapy benefit to patients, despite the trial's hypothesis that MPAs provide a superior framework for decision-making. Some patients who were shown the graphical representation of possible chemotherapy benefit interpreted this as definitive individual benefit for them, rather than the estimated population benefit these tools provide. This contradicted the ethos of the trial, i.e. examining the effectiveness of MPAs as predictors for individual patient benefit.

#### Addressing patients' preferences and expectations

Oncologists and research nurses anticipated that patient preferences would be a barrier to recruitment and often discussed this in terms of patients not wanting to forgo chemotherapy. The audio-recorded appointments showed that when preferences were expressed, recruiters tended to cease discussion about the trial; their accounts in interviews often indicated concern about upsetting the clinician–patient relationship.

*Well I think it's quite important not to [*respond to a patient preference*], it has the potential to kind of damage the relationship with the patient because the patient then feels, there's risk that they might feel they're letting you down.* (Oncologist Site 2)

In such instances, patients' perceptions (and potential misconceptions) about treatments were not addressed, resulting in missed opportunities to offer additional information patients might not have considered. For example, in one appointment the patient was concerned about hair loss from chemotherapy, although this only arose after she had declined the RCT in favour of endocrine therapy alone. The strategies that some other recruiters had used to try to inform patients about options available to address hair loss (e.g. cold capping, wigs) were not discussed in this instance.

A key issue that was reported in interviews and was apparent in some recordings was patients' up-front expectations for chemotherapy. Some patients reported that surgeons had referred to chemotherapy as advisable or the next step in their post-surgical treatment, reflecting the ‘signposting’ that might be offered in routine practice. In the context of OPTIMA Prelim, however, this could make it very difficult for oncologists to explain the rationale for the trial.

Relative: *‘Cos – I know, before today, my wife had decided that she wasn't going to go on the trial.*

Oncologist: *OK.*

Patient: *Um, purely because when I spoke to [Mr X, surgeon], my husband asked him what he'd do and he said if it was his sister, he would highly advise the chemo.*

### Phase 2: developing and delivering a ‘Plan of Action’ to optimise recruitment

Issues arising from phase 1 were first reported to the chief investigator/TMG 2 months after the QRI started. The QRI team worked with the TMG to co-produce a series of interventions to address recruitment challenges. Table 2 details the interventions and the timing of their implementation, together with the issues they were designed to address.

**Table 2:** OPTIMA Prelim recruitment challenges and corresponding QuinteT Recruitment Intervention (QRI)

QRI action implemented	Recruitment issue addressed
Tips and guidance document circulated to all sites (October 2013)	• Structuring the consultation • Explaining chemotherapy and its uncertainties • Talking about the test
Revised Patient Information Sheet distributed to all sites (October 2013)	• Convey more balanced portrayal of the advantages and disadvantages of test-directed chemotherapy
Group feedback: Scottish sites (November 2013) South West and Welsh sites (January 2014) Site 5 (March 2014) Site 6 (March 2014)	• Identifying potentially eligible patients • Approaching eligible patients • Discussing the study in recruitment appointments (conveying uncertainty of chemotherapy, use of chemotherapy predictor tools, explaining trial concepts, probing patient preferences)
Recruitment tips presented to newly opening sites (January 2014 onwards)	• Explaining chemotherapy and its uncertainties
Individual recruiter feedback to four oncologists (February to March 2014)	• Discussing the study in recruitment appointments (as above)

A ‘tips and guidance’ document was drafted and circulated to all sites and provided suggestions for structuring recruitment consultations, talking about the uncertain benefits of chemotherapy and discussing the Oncotype DX test (see Supplementary Material). The QRI researchers and TMG also revised the Patient Information Sheet to convey a clearer, more balanced portrayal of the advantages and disadvantages of using Oncotype DX to determine chemotherapy.

Individual and group feedback were core components of the plan of action, enabling the QRI team to use anonymised extracts from interviews and audio-recorded appointments to illustrate key recruitment issues and facilitate discussion around clinician-driven possible solutions. Extracts from the interviews and audio-recorded appointments illuminated variable views about eligibility criteria and patient preferences, and the chief investigator/TMG members contributed expert knowledge to elicit group discussion and engender peer-to-peer learning. At one session, the chief investigator used clinical vignettes portraying patients at the margins of the eligibility criteria to tease out and address sources of discomfort. Group feedback sessions also covered issues around structuring consultations, use of language and the subsequent ways in which these could influence patients' interpretation of trial information. In addition to these group sessions, oncologists who provided audio-recorded appointments received individual confidential feedback. Issues discussed included structuring the recruitment discussion by opening with chemotherapy uncertainty, explaining the study rationale and design, and strategies for clearly explaining the Oncotype DX test and RCT concepts.

### Recruitment outcomes

OPTIMA Prelim exceeded its recruitment target, consenting 350 patients and randomising 313 patients in 20 months against a target of 300 patients in 2 years. Having established its acceptability to patients and clinicians and secured funding, the main OPTIMA trial (with an integrated QRI) opened to recruitment in January 2017. The study is currently recruiting from more than 100 sites across the UK and Norway.

### Discussion

Our study used the QRI to understand and address the challenges of recruiting to a de-escalation trial comparing routine with a novel approach to determining adjuvant chemotherapy provision in patients with early stage breast cancer. Despite experiencing challenges, the recruitment target was eventually exceeded within the funded timescale through identifying and addressing the root causes of recruitment difficulties in real-time. We identified numerous ways in which oncologists' routine practices could either subtly or sometimes overtly inhibit RCT recruitment. There was evidence that some oncologists and MDTs gravitated towards offering the trial to a kernel of patients within the wider eligible population, avoiding those with clinical characteristics that they felt indicated a clear need for chemotherapy (e.g. greater lymph node involvement). When patients were approached, terminology and tools typically used to discuss chemotherapy in routine practice were found to contradict the rationale for OPTIMA Prelim and undermine recruitment. To tackle these challenges, a series of interventions were implemented, including a ‘tips and guidance’ document, individual and group feedback, and revisions to the Patient Information Sheet.

This study illuminated how oncologists' routine practices could inhibit recruitment at multiple stages of the recruitment process. There was evidence that not all clinicians and/or MDTs felt comfortable offering the study to all groups of eligible patients, which if widespread and inconsistently done across sites, might threaten the external validity of the definitive trial results as applied to all potentially eligible patients [22]. Discomfort with and selective application of trial eligibility criteria have been observed in other RCTs, as reported in a cross-trial investigation of recruitment issues across six RCTs that spanned different medical specialties (including oncology, mental health and surgery) [11,12]. Airing and addressing these issues was central to the OPTIMA Prelim ‘plan of action’. Recruiters were encouraged to consider their own perceptions of equipoise through clinical vignettes in group feedback sessions, where they had an opportunity to reflect on their practice and that of their peers and discuss and share concerns.

OPTIMA Prelim demonstrated the challenges of negotiating ‘current best practices’ in routine care with the endeavour of improving future patients' care through their role as trial recruiters. OPTIMA Prelim and the main trial have the potential to transform future practice, but this is counterbalanced by the possibility of significant consequences for current patients should the trial hypothesis not be supported. Furthermore, although accustomed to recruiting to trials exploring additional treatments, oncologists were less familiar recruiting to de-escalation trials [1,20,21,23,24]. A growing body of work has focussed on tensions recruiters face when trying to negotiate their ‘clinical’ and ‘research’ roles [11,12,22,25], contributing to the often ‘hidden’ emotional and intellectual challenges of recruiting patients [12,13]. Being mindful of the potential challenges and pitfalls of implementing trial eligibility criteria may help clinical investigators plan for and identify these issues more readily. Examining and reflecting on actual recruitment practices as a trial is underway is a fundamental part of the QRI and can provide a means of identifying and working through these trial-specific issues. Peer-to-peer interactions involving recruiters from different sites, as facilitated in OPTIMA Prelim through group feedback sessions, can be particularly helpful for engaging with these hidden challenges and dissolving discomforts.

OPTIMA Prelim recruitment, as with many oncology trials, relied on multidisciplinary professional involvement. MDT members collectively screened and identified potentially eligible patients. Breast surgeons and specialist nurses then had the important role of setting patients' expectations about adjuvant treatment before a full discussion about the study with the oncologist. Some surgeons continued to prepare eligible patients for adjuvant chemotherapy during post-surgery consultations, possibly reflecting a lack of awareness about the trial or equipoise issues. The importance of a collaborative approach to recruitment is well established [[26], [27], [28]], particularly in oncology [29], as is the drive for more holistic team approaches to recruitment training [[30], [31], [32], [33]]. Our findings from OPTIMA Prelim support an ongoing need to focus training and support team ownership of recruitment, especially with trials that span clinical disciplines.

The study had several limitations. Site participation in interviews and audio-recording of consultations was optional and nine of 25 sites participated. It is possible that participating sites represented more engaged clinicians/sites; nonetheless, it is notable that even these possibly more ‘engaged’ sites encountered the recurring challenges reported in this paper. It is anticipated that less engaged sites also encountered these issues, with possible other challenges not captured. Four of the 14 semi-structured interviews were conducted with TMG members: although valuable for providing insight into the motivation and expected conduct of the study, these interviewees may have skewed the findings, although the issues reported in this paper were reflective of the general, recurring issues that arose across the dataset.

Conversely, strengths of the study included the use of audio-recordings of real recruitment interactions to examine what actually happens during recruitment discussions (rather than relying solely on informants' reported practices) and the triangulation of multiple methods to identify the key sources of recruitment difficulty. This enabled a detailed and robust understanding of recruitment issues in a relatively short space of time, which allowed the trial team to develop and implement strategies to address issues as the trial was underway (rather than towards the end of an RCT, when it is often too late). The integration of multiple methods and converging findings lends credibility to our core findings, although future research should build on this study to examine whether these novel insights are relevant in a wider array of RCTs. Further research should also be invested into the best methods of overcoming the common challenges recruiters face, including the cost-effectiveness of training and feedback interventions, such as the QRI.

## Conclusion

This was the first study to expose the challenges of negotiating ‘routine clinical practice’ with clinical trial recruitment, which is now a core aspect of practising clinicians' roles. The findings highlight the need to support clinicians to negotiate their clinical and research roles, particularly in the context of de-escalation cancer trials.

## Conflict of Interest

I. Macpherson has received speaker fees from Genomic Health.

## Footnotes

1 National Cancer Registration and Analysis ServiceEast Regional Office, Victoria House, Capital Park, Fulbourn, Cambridge CB21 5XB. Email: info@predict.nhs.uk.

2 Online – Ajuvant! Inc, San Antonio, TX, USA.
