# Supplementary material for: Strategies to Improve Recruitment to a De-escalation Trial: A Mixed-Methods Study of the OPTIMA Prelim Trial in Early Breast Cancer
Source: Clin Oncol (R Coll Radiol). 2020 Jun;32(6):382–9. doi: 10.1016/j.clon.2020.01.029 (PMC7246331; doi:10.1016/j.clon.2020.01.029)
Supplement: Multimedia component 1 [file mmc1.docx]

#### **OPTIMA Prelim Study Design and History**

| OPTIMA prelim was designed to demonstrate the feasibility of a large-scale UK-based RCT of multi -parameter assay (MPA) directed chemotherapy and to select a test to be used in that trial. OPTIMA prelim recruited women aged 40 or older with resected ER-positive, HER-2 negative breast cancer, who had either 1-9 involved axillary nodes or a tumour of at least 30mm size. Randomisation was on a 1:1 ratio to current management (chemotherapy followed by endocrine therapy) or to MPA directed treatment using Oncotype DX. Those with a Recurrence Score of >25 (defined by the manufacturer as “high risk”) received chemotherapy followed by endocrine therapy whilst those with a score ≤25 (“intermediate/low risk”) received endocrine therapy alone. The trial was partially blinded, in that the randomised allocation was not disclosed to patients or the treating clinician. However, blinding was not possible where patients were randomised to the test-directed arm and received a ‘no chemotherapy’ outcome. The process of consent and Oncotype DX testing took approximately three weeks, but this short delay to treatment was not considered detrimental to a patient’s outcome.  The first OPTIMA prelim participant was recruited in October 2012 and the study closed to recruitment in August 2014. In total, 35 hospitals from 31 NHS trusts and health boards in England, Scotland and Wales participated. Most recruitment took place before Oncotype DX testing was routinely available in the NHS. NICE recommended the Oncotype DX test be made available to some patients with node negative disease in September 2013 [34]. However, it was only in April 2014 that the recommendation started to be implemented in England; it took longer in Wales and Scotland. Although the Oncotype DX test had been available in private healthcare for some time, familiarity with the test would have been limited during most of the recruitment period.  Trial outcomes included achieving recruitment of 300 patients within 2 years from the first site opening, recruitment of the final 150 patients within a 6-month period and a patient acceptance rate of at least 40%. These outcomes were met on 3 June 2014 when at the time of database lock, 350 patients had consented to join the study and 313 had proceeded to randomisation. Reasons for non-randomisation included central confirmation of receptor status or Oncotype DX testing still in progress (n=15), ineligibility (n=15) [which included discrepant receptor status on central re-testing (n=12)] and pre-randomisation withdrawal by either patient or clinician (n=7).  OPTIMA prelim also included an evaluation of alternative MPAs to determine which technology should be evaluated in the main trial[35, 36]. Prosigna was selected as the MPA for use in the OPTIMA main. |
| --- |

34. National Institute for Health and Care Excellence *Gene expression profiling and expanded immunohistochemistry test for guiding adjuvant chemotherapy decisions in early breast cancer management: MammaPrint, Oncotype DX, IHC4 and Mammostrat*, in *Diagnostics*

*Guidance [DG10]*. 2013

35. Hall, P.S., A. Smith, C. Hulme, A. Vargas-Palacios, A. Makris, L. Hughes-Davies, et al., *Value of Information Analysis of Multiparameter Tests for Chemotherapy in Early Breast Cancer: The OPTIMA Prelim Trial.* Value Health, 2017. **20**(10): p. 1311-1318.

36. Bartlett, J.M.S., J. Bayani, A. Marshall, J.A. Dunn, A. Campbell, C. Cunningham, et al., *Comparing Breast Cancer Multiparameter Tests n the OPTIMA Prelim Trial: No Test Is More Equal Than the Others.* Journal of the National Cancer Institute, 2016. **108**(9).

# OPTIMA study: guidance for recruiters, September 2013

**This document lists suggestions that may help recruiters during appointments. You may wish to consider including some of these suggestions in your appointments, alongside your own individual approach and style of discussing the OPTIMA study with patients.**

**Starting the appointment**

- Patients often find it reassuring to hear a summary of their surgery/pathology results
- It is helpful to mention the OPTIMA study briefly early on

**Information provision: different types of breast cancer and individualised/personalised treatment**

- It is helpful to recount how knowledge of breast cancer has evolved over past decade with a range of treatment options in their clinical situation, and emphasise how we are constantly trying to improve treatment: e.g. tailoring a treatment plan to individual patients and types of cancer.

**Purpose and overview of OPTIMA**

- The aim of the study is to find out whether a personalised decision about whether to have chemotherapy or not can be made safely and effectively using a test such as Oncotype DX.
- All patients receive hormone therapy because this is known to prevent future recurrence. It has fewer side effects than chemotherapy.
- Chemotherapy would usually also be given but recent research has indicated that many women with this type of breast cancer may not benefit from it.
- Usually the decision about chemotherapy is based on the size and grade of the tumour and the number of lymph glands involved. However, these tests are not very sensitive.
- Tests such as Oncotype DX have been developed to try to better predict who would benefit from chemotherapy and who could avoid having it, by providing better information about the nature of the tumour and how it is likely to behave.
- Explain what chemotherapy entails and its side effects. You might want to use these words, adding the particular patient’s level of risk instead of ‘x’:

*“We would estimate that for a patient like you, there is an x% chance that the chemo will stop the cancer from coming back. This means that if there were 100 people treated with chemo, only x would benefit from it, but all are likely to experience the side effects.”*

- You might want to explain that as we currently do not know how to predict who will benefit from chemotherapy, most women are advised to have it even though we know many will not be able to benefit but will experience the side effects.
- The aim of the OPTIMA study is to enable us to gain evidence to enable us to be better at targeting chemotherapy to women who will benefit from it, and help women who will not benefit from going through an unnecessary treatment with side effects.

**Introducing the OPTIMA study and the Oncotype DX test**

- Explain that the ODx test is used in the US and other countries, but is not yet recommended for use in the NHS.
- In the OPTIMA study we want to include women with larger tumours and those that have spread to the lymph glands, as well as in those with smaller, more confined tumours - to evaluate how useful it can be.
- It is good to mention that OPTIMA is a study funded by the National Institute for Health Research (the NHS funding body) and is being carried out around the UK.
- It is also important to explain that the doctors and nurses involved in the patient’s care have already discussed their specific case, and agreed that it would be appropriate to offer them the opportunity to take part in the trial. They are suitable to receive either of the study’s two treatment options.

**Explaining the OPTIMA study**

- When you explain the study design, you might like to draw the treatment options (arms) on a piece of paper, or use a pre-prepared diagram – such as the one in the Patient Information Sheet.
- Make sure you call the arms ‘treatment option 1’ and ‘treatment option 2’. Try to avoid calling them ‘standard’ or ‘experimental’
- Explain that option 1 is what they would receive if they do not take part in OPTIMA – hormone therapy and chemotherapy. Option 2 is determined by the Oncotype DX test – that a sample of the tumour tissue (already removed during surgery) will be tested and the result will determine whether they will be recommended to have chemotherapy in addition to hormone therapy.
- You need to explain how they will be allocated to option 1 or option 2. We have found, from previous trials, that an explanation something like this can work well:

*“All women who agree to take part in the OPTIMA study will be allocated to option 1 or option 2 through a process called ‘randomisation’. This means that you will be assigned to option 1 or option 2 by chance – that you cannot choose and your doctor cannot choose. This is so that the options can be compared fairly – that the two groups will contain similar numbers and will be as similar to each other in all other respects.*

- It is important to re-iterate that the patient is suitable to receive either of the study’s two treatment options, and that everyone will receive hormone therapy. Also that all patients will be followed up very carefully – every three months in the first year, and once a year after that.
- It is also important to explain how and why the patient and doctor will not know whether the patient was allocated to option 1 or option 2. We have found that the following can work well:

*“Neither you nor the doctors will be told whether you were in option 1 or 2. This is because we need to compare these two groups as fairly as possible. Our results will be more reliable if no other factors influence us and we can be more confident that the patients and doctors are behaving the same ways if they do not know whether the patient is in option 1 or 2. There is one exception to this: if you went into the test group and were recommended not to have chemotherapy, we would know that this must be because you have had the test done, and came back not needing chemotherapy. Looking back at this diagram, we would know this, because all the other groups receive chemotherapy as well as hormone therapy.”*

**Explaining timing and follow up within OPTIMA**

- It is important to explain that patients taking part in OPTIMA will need to wait 3-4 weeks before starting treatment, regardless of whether they are in option 1 or option 2 because of the need to test the tissue in the special laboratory (use diagram).
- It is even more important to reassure the patient that waiting this amount of time will NOT have any detrimental effects on their health or outcome. For most, the cancer will have been present for some time and so this short delay will not make any difference. Chemotherapy will be booked for all patients immediately because it takes two weeks to organise. If they then do not need chemotherapy, it will be cancelled.

**Closing the appointment**

- Reassure patient that they can take some time to consider whether they want to take part. Make sure they have the main OPTIMA information sheet. Advise them that can contact the research nurse [or whoever appropriate] if they have questions.
- Explain audio recording; give QRS information sheet/consent form.
